# Pharmacological Inhibition of STAT3 by Stattic Ameliorates Clinical Symptoms and Reduces Autoinflammation in Myeloid, Lymphoid, and Neuronal Tissue Compartments in Relapsing–Remitting Model of Experimental Autoimmune Encephalomyelitis in SJL/J Mice

**DOI:** 10.3390/pharmaceutics13070925

**Published:** 2021-06-22

**Authors:** Khalid Alhazzani, Sheikh F. Ahmad, Naif O. Al-Harbi, Sabry M. Attia, Saleh A. Bakheet, Wedad Sarawi, Saleh A. Alqarni, Mohammad Algahtani, Ahmed Nadeem

**Affiliations:** Department of Pharmacology and Toxicology, College of Pharmacy, King Saud University, Riyadh 11451, Saudi Arabia; kalhazzani@ksu.edu.sa (K.A.); fashaikh@ksu.edu.sa (S.F.A.); nharbi1@ksu.edu.sa (N.O.A.-H.); attiasm@ksu.edu.sa (S.M.A.); sbakheet@ksu.edu.sa (S.A.B.); wsarawi@ksu.edu.sa (W.S.); salqarni444@gmail.com (S.A.A.); mohmalgahtani@ksu.edu.sa (M.A.)

**Keywords:** multiple sclerosis, granulocytes, CD4+ T cells, STAT3 inhibition, IL-17A, iNOS

## Abstract

Multiple sclerosis (MS) is an immune-mediated inflammatory disease that leads to demyelination and neuronal loss in the central nervous system. Immune cells of lymphoid and myeloid origin play a significant role in the initiation and amplification of neuronal inflammation in MS. STAT3 signaling plays a pivotal role in both myeloid and lymphoid immune cells, such as neutrophils and CD4+ T cells, through regulation of their inflammatory potential. Dysregulation in STAT3 signaling in myeloid and lymphoid cell compartments has been reported in MS. In this report, we attempted to investigate the effect of a small molecular inhibitor of STAT3, i.e., Stattic, in a relapsing–remitting (RR) model of experimental autoimmune encephalomyelitis (EAE). The effect of Stattic was investigated for clinical features, oxidative stress parameters, and Th17-related signaling in both the periphery and brain of SJL/J mice. Our data report that p-STAT3 expression is elevated in granulocytes, CD4+ T cells, and brain tissue in myelin proteolipid protein (PLP)-immunized SJL/J mice, which is associated with the presence of clinical symptoms and upregulation of inflammatory markers in these cells/tissues. Treatment with Stattic leads to the amelioration of disease symptoms and attenuation of inflammatory markers in neutrophils (iNOS/nitrotyrosine/IL-1β), CD4+ T cells (IL-17A/IL-23R), and brain tissue (IL-17A/iNOS/IL-1β/MPO activity/lipid peroxides) in mice with EAE. These data suggest that the blockade of STAT3 signaling in cells of lymphoid and myeloid origin may cause the attenuation of systemic and neuronal inflammation, which could be responsible for the amelioration of disease symptoms in an RR model of EAE. Therefore, pharmacological inhibition of STAT3 in RRMS could be a potential therapeutic strategy.

## 1. Introduction

Multiple sclerosis (MS) is an immune-mediated neuroinflammatory disorder that is responsible for causing serious physical disability in 2.5 million people worldwide, especially young adults [1]. MS involves confluent demyelination in the central nervous system (CNS), i.e., the brain and spinal cord, which leads to plaque formation and neuronal loss. Loss of myelin sheaths and oligodendrocytes in the CNS causes neurological impairment, which includes impaired cognition, visual difficulties, limb weakness, and ataxia. Demyelinating plaques sometimes appear even before the manifestation of clinical symptoms and may cause blood–brain barrier (BBB) impairment [1,2,3].

The immunopathogenesis of MS is extraordinarily complex in that immune cells of both myeloid and lymphoid origin play a crucial role, which is also corroborated by large-scale genetic studies [4,5,6]. Several prior studies have shown the compelling role of neutrophils, macrophages/dendritic cells (DCs), and T cells in the initiation and progression of MS. For example, leukocytes from peripheral blood are reported to display a higher expression of genes related to inflammation and antigen processing/innate immune responses in relapsing–remitting/progressive MS patients [4,5]. These immune cells have also been shown to be activated in peripheral circulation, as well as demyelinating plaques in the brains of MS patients [1,6].

Myeloid immune cells serve critical roles in various autoimmune/inflammatory disorders, including MS [7,8,9]. STAT3 signaling is reported to be critical in the regulation of myeloid cell function. Immune cells of myeloid origin are reported to elevate STAT3 levels in the brains of MS patients [10]. MS patients are also reported to have upregulated levels of p-STAT3 in the peripheral circulation and the brain [10,11]. Similarly, mice with EAE have increased p-STAT3 levels in immune cells of myeloid origin [10]. Elevated STAT3 levels may be associated with increased expression/release of pro-inflammatory cytokines, as well as other mediators, which include IL-1β, iNOS, MPO, and MMP [9,12].

STAT3 signaling is also utilized by several inflammatory cytokines that signal through cell surface receptors on T cells [13]. STAT3 activation is required for polarization of Th0 (CD4+ T cells) into Th17 cells (IL-17-producing CD4+ T cells) in the presence of appropriate signals [14]. These signals originate from DCs or other antigen-presenting cells in the form of cytokines such as IL-6/IL-23 and costimulatory molecules [15]. Th17 cells hugely influence the development and progression of MS both in human subjects and mice models of EAE through the release of signature cytokines such as IL-17A/IL-22 [6,12,16,17]. Thus, the STAT3 pathway also serves as a bridge between innate and adaptive immune responses and is crucial to the overall maintenance of immune homeostasis.

The STAT3 pathway is reported to be activated in several immune-mediated inflammatory diseases, such as rheumatoid arthritis, lupus, asthma, and MS. There is enough evidence that the aberrant activation of STAT3 has pathological consequences in MS patients and EAE mice. Knocking out STAT3 in CD4+ T cells or myeloid cells has been reported to cause an amelioration in disease symptoms and a reduction in inflammatory markers in the periphery/brain [10,18].

Several studies have used small-molecule STAT3 inhibitors, such as STA-21/C188-9/Stattic, to suppress STAT3 signaling in different animal models of autoimmune/inflammatory diseases, such as asthma, lupus nephritis, and arthritis [19,20,21]. These studies show the feasibility of using a STAT3 inhibition approach to mitigate autoimmune inflammation. However, a small molecule pharmacological inhibition approach to target STAT3 has not been investigated in a relapsing–remitting model of EAE.

A relapsing–remitting (RR) model of experimental autoimmune encephalomyelitis (EAE) in SJL/J mice is a commonly used model that mimics several features of RRMS in humans, as it involves remission and relapse on its own [22]. Further, immune cells of both myeloid and lymphoid origin play a critical role in disease induction and progression. Furthermore, the presence of demyelinating plaques in the CNS of EAE mice results from inflammatory mediators being released by neutrophils, lymphocytes, DCs, macrophages, and microglia [1,23,24]. Ultimately, EAE is driven by interactions between autoreactive Th17/Th1 cells and neutrophils/DCs that amplify and perpetuate local neuroinflammatory autoimmune reactions [1,6,17,23]. Therefore, this mouse model of RRMS provides great potential to test the efficacy of a therapeutic compound in a preclinical setting.

Therefore, the focus of this investigative report was to study the effect of the STAT3 signaling blocker Stattic on clinical symptoms and inflammatory mediators in the brain and peripheral myeloid/lymphoid immune cells. Our study shows that an activated form of STAT3, i.e., phosphorylated STAT3 (p-STAT3), is elevated in neutrophils, CD4+ T cells, and neuronal tissue, which is associated with increased neuronal/peripheral inflammation and disease symptoms in an RR model of EAE. Stattic causes the amelioration of disease symptoms through a reduction in p-STAT3 levels and peripheral/neuronal inflammation in mice with EAE.

## 2. Materials and Methods

### 2.1. Animals

The SJL/J mice used in this study were procured from Jackson Laboratories (Bar Harbor, ME, USA) and housed at the Animal Facility of the College of Pharmacy, King Saud University. Female mice were used with ages ranging from 7 to 8 weeks. Mice were habituated to the surrounding temperature, humidity, and circadian rhythm (24–26 °C with 60% humidity and 12 h light/12 h dark cycle) before the start of the immunization process and were provided with food and water ad libitum. The protocols required for carrying out the experiments were approved by the Institutional Animal Care and Use Committee, King Saud University.

### 2.2. Development of Relapsing–Remitting (RR) Experimental Autoimmune Encephalomyelitis (EAE) Model in SJL/J Mice

For the induction of RR type of EAE in SJL/J mice, mice were immunized with 200 µg of myelin proteolipid protein 139-151 (PLP139-151) peptide emulsified in CFA, which was procured from Hooke Laboratories (USA). Mice were also administered 200 ng of pertussis toxin (Hooke Laboratories, USA) intraperitoneally (i.p.) on the same day to activate innate immunity. Evaluation of clinical symptoms of EAE was carried out according to the following criteria: (0) no disease symptoms, (1) complete paralysis of the tail, (2) partial hind weakness/paralysis, (3) complete hind limb paralysis, (4) front and hind limb paralysis, and (5) moribund state. If animals reached a clinical score of 5, they were sacrificed and eliminated from the study.

### 2.3. Experimental Groups

To evaluate the role of STAT3 signaling in neuronal inflammation in SJL/J mice, they were administered Stattic at a dose of 10 mg/kg i.p. (Tocris, Bristol, UK) or vehicle every alternate day for a period of 20 days from day 10 post-immunization. Stattic was used at a dose of 10 mg/kg, as this dose has been previously reported to suppress p-STAT3 levels in a progressive nephritis model in mice [25]. After the appearance of disease symptoms, SJL/J mice were randomly allocated on day 10 into one of the following groups: Group A, vehicle-administered control SJL/J mice (Veh + Control SJL/J): SJL/J mice (non-diseased mice) were administered only drug vehicle. Group B, drug-administered control SJL/J mice (Stattic + Control SJL/J): SJL/J mice (non-diseased mice) were administered Stattic at a dose of 10 mg/kg i.p. every alternate day for a period of 20 days, as written above. Group C, vehicle-administered SJL/J mice with EAE (Veh + EAE SJL/J): SJL/J mice were administered with PLP for immunization and drug vehicle, as written above. Group D, drug-administered SJL/J mice with EAE (Stattic+EAE SJL/J): SJL/J mice were administered with PLP for immunization and Stattic at a dose of 10 mg/kg i.p. every alternate day for a period of 20 days, as written above. Mice were sacrificed at the end of the treatment period by deep inhalational anesthesia, and different tissues (brain/spleen) were extracted for various biochemical and molecular analyses.

### 2.4. Real-Time PCR

The cerebral cortex was isolated in RNAlater and kept at −20 °C for a month before being used for the isolation of RNA. Total RNA was extracted from these samples utilizing RNeasy mini kit (Qiagen, Germantown, MD, USA), followed by transformation of 0.5 μg RNA into cDNA through reverse transcription technique (High-Capacity cDNA archive kit, Applied Biosystems, Grand Island, NY, USA), according to the directions of the manufacturer [26,27]. mRNA expression of IL-17A, IL-1β, and iNOS was evaluated by real-time PCR on ABI PRISM 7500 sequence detection system (Applied Biosystems), as stated before [26,27]. Fold change in mRNA expression of different markers was analyzed by a well-known method of relative gene expression analysis, as described before [28].

### 2.5. Flow Cytometry of Cell Surface/Intracellular Markers in Splenocytes

Spleens were isolated from different groups and crushed by a 2 mL syringe plunger on a 100 µm sieve, followed by sieving on a 70 µm strainer to obtain a single cell suspension in RPMI-1640, as stated before [26,27,29]. After lysis of RBCs, splenocytes were immunostained for cell surface markers such as CD4/GR-1 (BioLegend, San Diego, CA, USA) using monoclonal antibodies fluorescently coupled to FITC/PE-Dazzle/APC-Cy7/APC. After the normal procedure of fixation and permeabilization of splenic leukocytes (Miltenyi Biotech, Bergisch Gladbach, North Rhine-Westphalia, Germany), cells were then immunolabeled for intracellular proteins such as p-STAT3, IL-17A, IL-23R, IL-1β, iNOS, and nitrotyrosine (Santacruz Biotech, Dallas, Texas, USA; BioLegend, San Diego, CA, USA; R&D Systems, Minneapolis, MN, USA) using monoclonal antibodies fluorescently coupled to FITC/PE/APC. Splenic leukocytes immunostained with cell surface and intracellular proteins were then acquired on a flow cytometer (Beckman Coulter, Indianapolis, IN, USA), followed by an evaluation of the 10,000 events (cells) according to the fluorescent characteristics (Supplementary Figure S1) using Cytomics FC 500 software, as stated earlier [26,27,29].

### 2.6. Evaluation of Inflammatory Cytokines in the Brain by ELISA

The assessment of IL-17A/IL-1β protein levels in the cerebral cortex was conducted utilizing ELISA kits (R&D Systems, USA; Biolegend, USA) according to the instructions of the supplier (Supplementary Materials). Data are displayed as pg/mg protein.

### 2.7. Evaluation of Myeloperoxidase (MPO) Activity and Lipid Peroxides in the Brain

MPO activity in the cerebral cortex as a measure of neutrophilic inflammation was assessed, as described before [29,30]. Lipid peroxides were measured in the cerebral cortex, as described earlier [29].

### 2.8. Evaluation of p-STAT3 Levels in the Brain by ELISA

Assessment of protein levels of p-STAT3 at Tyr705 (Pathscan^®^ Phospho-STAT3) in the cerebral cortex was conducted by an ELISA kit (Cell Signaling Technology, Danvers, MA, USA), as recommended by the guidelines of the supplier and as stated previously [30].

### 2.9. Chemicals and Reagents

Chemicals/reagents in this investigation were of the highest quality and were procured from Sigma Chemicals (St. Louis, MO, USA) if not stated otherwise.

### 2.10. Statistical Analysis

The results are displayed as mean ± SEM. Comparisons among different groups for the investigated parameters were carried out by ANOVA (analysis of variance), followed by Tukey’s multiple comparison tests. The area under the curve (AUC) was calculated to assess overall disease severity in Stattic-treated and vehicle-treated EAE groups, and these two groups were compared by an unpaired *t*-test. Results were considered statistically significant if *p* < 0.05. All statistical analyses were conducted using Graphpad Prism 9 (San Diego, CA, USA).

## 3. Results

### 3.1. Blockade of STAT3 Signaling Improves Clinical Features Related to EAE in PLP-Immunized SJL/J Mice

**Figure 1:**
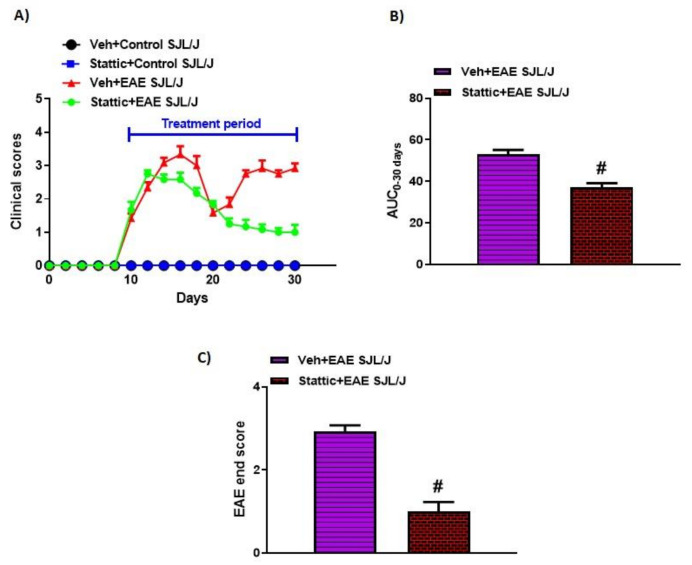
Pharmacological targeting of STAT3 pathway by small-molecule inhibitor Stattic ameliorates clinical symptoms of EAE in SJL/J mice: (**A**) Clinical scores during the experiment; (**B**) AUC from day 0 to 30; (**C**) clinical scores at the end of the study. Data are expressed as mean ± SEM, *n* = 6–8/group. # *p* < 0.05 vs. Veh + EAE SJL/J group.

Past studies have shown that various inflammatory cytokines signal via STAT3 both in innate and in adaptive immune cells. Therefore, in this study, we attempted to explore whether a pharmacological inhibition approach using a small molecule to target STAT3 would modulate disease features in an RR model of EAE in SJL/J mice. Our data show that PLP-immunized SJL/J mice developed classic features of RRMS, as depicted by remission and relapse in different phases. These mice had tail paralysis and hind limb weakness/partial paralysis ascending to forelimbs, which ameliorated itself during the remission phase. Treatment with the small-molecule inhibitor of STAT3, Stattic, after onset of the disease led to the amelioration of disease symptoms during the first relapse phase, which continued until the second relapse phase (Figure 1A). This was confirmed by a reduction in end clinical scores and AUC0-30 days in Stattic-treated EAE mice compared to vehicle-treated EAE mice (Figure 1B,C). These data depict that pharmacological inhibition of STAT3 by small-molecule Stattic is useful in treating disease symptoms in an RR model of EAE in SJL/J mice.

### 3.2. Upregulation of Activated STAT3/Inflammatory Mediators and Its Attenuation by Stattic in Innate Immune Cells of PLP-Immunized SJL/J Mice

**Figure 2:**
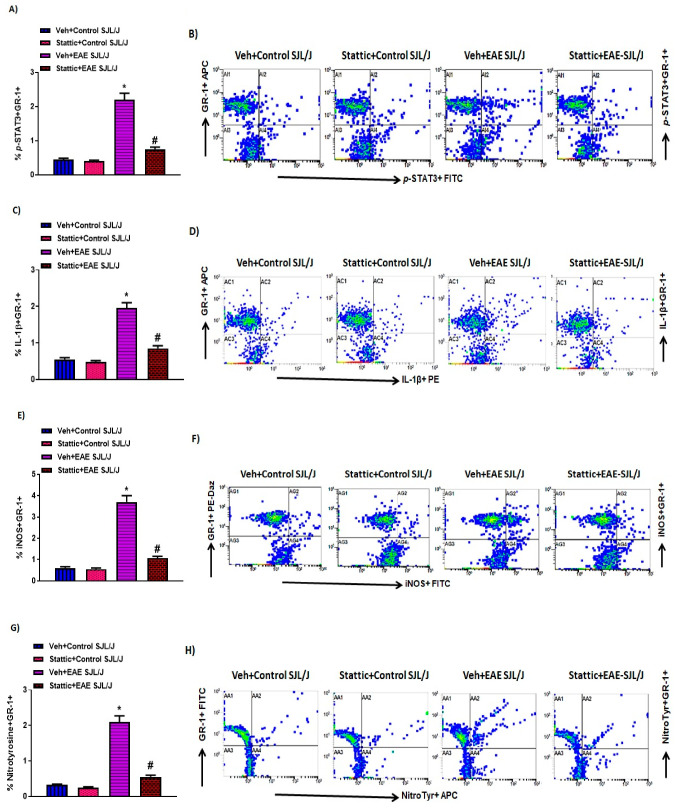
Elevated STAT3 signaling in neutrophils and associated oxidative inflammation, and its inhibition by small-molecule pharmacological inhibitor Stattic in SJL/J mice with EAE: (**A**) % of p-STAT3+ GR-1+ cells. (**B**) An illustrative flow plot displaying immunostaining of p-STAT3+ and GR-1+ cells. (**C**) % of IL-1β+ GR-1+ cells. (**D**) An illustrative flow plot displaying immunostaining of IL-1β+ and GR-1+ cells. (**E**) % of iNOS+ GR-1+. (**F**) An illustrative flow plot displaying immunostaining of iNOS+ and GR-1+ cells. (**G**) % of Nitrotyrosine+ GR-1+. (**H**) An illustrative flow plot displaying immunostaining of Nitrotyrosine+ and GR-1+ cells. Data are expressed as mean ± SEM, *n* = 6/group. * *p* < 0.05 vs. Veh + Control SJL/J group/ Stattic + Control SJL/J group; # *p* < 0.05 vs. Veh + EAE SJL/J group.

It has been previously investigated that STAT3 plays a crucial role in myeloid cells, such as neutrophils. Therefore, in this study, we wanted to investigate whether there are changes in the activated form of STAT3, i.e., p-STAT3 levels, in these immune cells during active disease in an RR model of EAE in SJL/J mice. Our data reveal that neutrophils have increased p-STAT3 levels in PLP-immunized mice (Figure 2A,B). Further, the administration of Stattic, a STAT3 signaling inhibitor, caused significant attenuation in p-STAT3 expression in these innate immune cells (Figure 2A,B). Our next goal was to track the inflammatory mediators associated with these innate immune cells, i.e., iNOS, nitrotyrosine, and IL-β. Our data show that iNOS+, nitrotyrosine+, and IL-β+ immunostaining increased in granulocytes, i.e., GR-1+ cells (Figure 2C–H). Further, iNOS+, nitrotyrosine+, and IL-β+ expressing GR-1+ cells were significantly decreased by treatment with the STAT3 blocker Stattic in PLP-immunized SJL/J mice (Figure 2C–H). These data depict that STAT3 activation plays a significant role in the inflammatory signaling of myeloid cells (granulocytes) in PLP-immunized SJL/J mice. This is corroborated by a significant reduction in inflammatory mediators of granulocytes by Stattic administration in EAE mice.

### 3.3. Upregulation of Activated STAT3/Inflammatory Mediators and Its Attenuation by Stattic in CD4+ T Cells in PLP-Immunized SJL/J Mice

**Figure 3:**
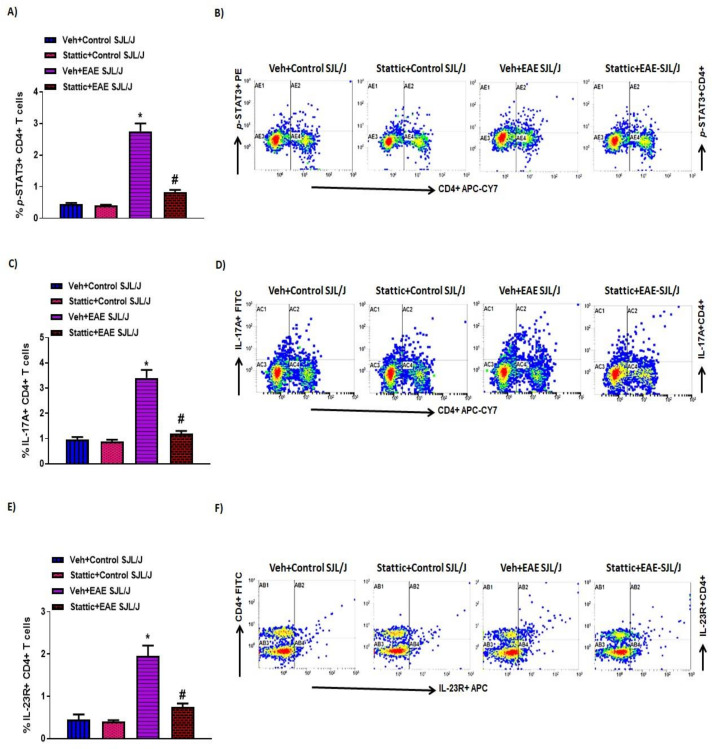
Elevated STAT3 signaling in CD4+ T cells and associated inflammation, and its inhibition by small-molecule pharmacological inhibitor Stattic in SJL/J mice with EAE: (**A**) % of p-STAT3+ CD4+ T cells. (**B**) An illustrative flow plot displaying immunostaining of p-STAT3+ and CD4+ T cells. (**C**) % of IL-17A+ CD4+ T cells. (**D**) An illustrative flow plot displaying immunostaining of IL-17A+ and CD4+ T cells. (**E**) % of IL-23R+ CD4+ T cells. (**F**) An illustrative flow plot displaying immunostaining of IL-23R+ and CD4+ T cells. Data are expressed as mean ± SEM, *n* = 6/group. * *p* < 0.05 vs. Veh + Control SJL/J group/Stattic + Control SJL/J group; # *p* < 0.05 vs. Veh + EAE SJL/J group.

STAT3 signaling is also critical in CD4+ T cells, as it helps in the differentiation of Th17 cells, which are known to play an important role in MS. Therefore, in this study, we also investigated whether there are changes in the activated form of STAT3, i.e., p-STAT3 levels, in CD4+ T cells during the active phase of the disease in PLP-immunized SJL/J mice. Our data show that CD4+ T cells have increased p-STAT3 levels in PLP-administered mice (Figure 3A,B). Further administration of Stattic, a STAT3 signaling inhibitor, led to significant attenuation in p-STAT3 in CD4+ T cells (Figure 3A,B). Our next objective was to assess Th17-related inflammatory mediators, i.e., IL-17A and IL-23R, in PLP-immunized mice. Our data show that IL-17A+ and IL-23R+ immunostaining increased in the CD4+ T cells of PLP-immunized SJL/J mice (Figure 3C–F). Further, IL-17A+ and IL-23R+ immunostaining in CD4+ T cells was significantly downregulated by treatment with the STAT3 blocker Stattic in PLP-immunized SJL/J mice (Figure 3C–F). These data display that STAT3 activation also serves a crucial role in the inflammatory signaling of Th17 cells in PLP-immunized SJL/J mice. This is confirmed by a significant reduction in Th17-related inflammatory mediators in SJL/J mice by Stattic administration.

### 3.4. Upregulation of Activated STAT3, and Inflammatory/Oxidative Mediators, and Its Attenuation by Stattic in CNS of PLP-Immunized SJL/J Mice

**Figure 4:**
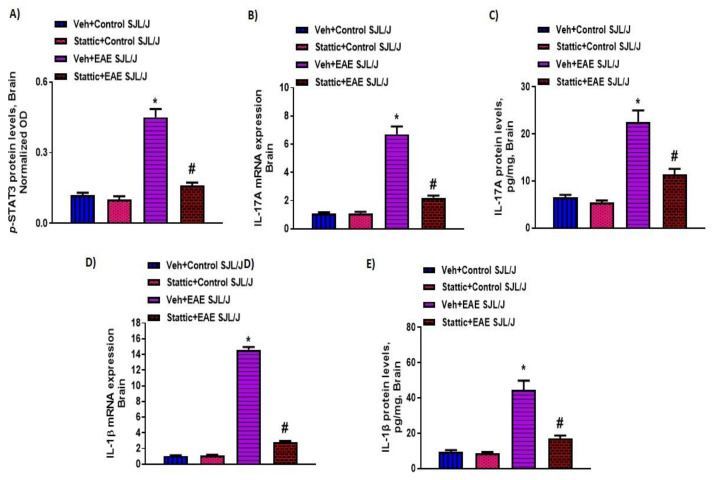
Elevated STAT3 signaling in the CNS and associated inflammation, and its inhibition by small-molecule pharmacological inhibitor Stattic in SJL/J mice with EAE: (**A**) p-STAT3 protein levels; (**B**) IL-17A mRNA expression; (**C**) IL-17A protein levels; (**D**) IL-1β mRNA expression; (**E**) IL-1β protein levels. Data are expressed as mean ± SEM, *n* = 6–8/group. * *p* < 0.05 vs. Veh + Control SJL/J group/ Stattic + Control SJL/J group; # *p* < 0.05 vs. Veh + EAE SJL/J group.

**Figure 5:**
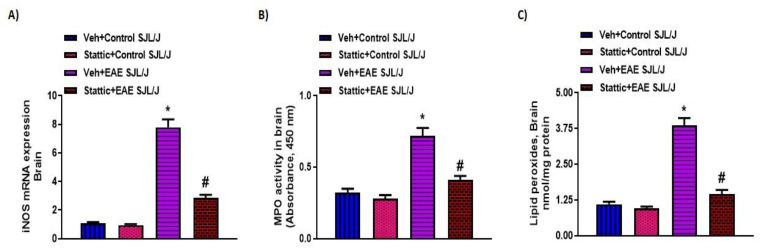
Oxidative inflammation in the CNS and its inhibition by small-molecule pharmacological inhibitor Stattic in SJL/J mice with EAE: (**A**) iNOS mRNA expression; (**B**) MPO activity; (**C**) lipid peroxides. Data are expressed as mean ± SEM, *n* = 6–8/group. * *p* < 0.05 vs. Veh + Control SJL/J group/ Stattic + Control SJL/J group; # *p* < 0.05 vs. Veh + EAE SJL/J group.

Lastly, we corroborated whether STAT3 signaling was dysregulated in the CNS of PLP-immunized mice and whether it was associated with neuroinflammatory changes. Our results indicate that p-STAT3 levels (Figure 4A) were upregulated in the CNS of PLP-immunized SJL/J mice, which were linked with increased Th17-related inflammatory markers, i.e., mRNA and protein levels of IL-17A and IL-1β (Figure 4B–E). Increased p-STAT3 levels in the brain were also associated with increased oxidative parameters such as iNOS mRNA levels, as well as MPO activity and lipid peroxides (Figure 5A–C). Next, we explored whether neuronal inflammation was affected by Stattic administration in PLP-immunized mice. Our data show that treatment with Stattic led to a reduction in levels of inflammatory cytokines and oxidative mediators, i.e., IL-17A, IL-1β, MPO, iNOS, and lipid peroxides, in mice with EAE (Figure 4 and Figure 5). These data suggest that Stattic acts at multiple cells/tissues such as myeloid, lymphoid, and CNS to ameliorate disease symptoms in an RR model of EAE in SJL/J mice.

## 4. Discussion

MS is an autoimmune and neuroinflammatory disease that leads to demyelination and axonal loss in the CNS and spinal cord. Multiple interactions between the myeloid and lymphoid immune cells play a critical defensive function in normal situations; however, when autoreactivity develops due to the breakage of self-tolerance, it leads to neuronal inflammation [1,8,31]. T cells, neutrophils, and macrophages contribute to neuroinflammation and axonal loss [3,9]. Myeloid cells such as neutrophils/DCs help in the presentation of autoantigens, which propels autoreactivity in T/B cell compartments [3,31,32]. Thus, both innate and adaptive immune cells contribute to MS pathology in which STAT3 signaling may play a crucial role. Our study reveals that STAT3 signaling is elevated both in lymphoid and myeloid cell compartments and is linked with inflammation in the brain of an RR mouse model of EAE. Furthermore, STAT3 inhibition by Stattic causes an improvement in clinical symptoms in an RR model of EAE.

STAT3 signaling serves a crucial function in the regulation of immune responses of both adaptive and innate immune cells. The dysregulation of STAT3 signaling has been reported to be linked with many autoimmune and inflammatory diseases, including MS [14,33,34]. The STAT3 gene has also been reported to be associated with MS susceptibility [35,36]. Hyperactivation of STAT3 is also reported to be elevated in the peripheral blood of MS patients and EAE mice models [11,34,37,38].

STAT3 inhibitors have demonstrated preclinical efficacy in an animal model of osteoarthritis, lupus, and other inflammatory diseases [19,20,39]. STAT3 knockout in CD4+ T cells has shown reduced clinical symptoms in EAE mice. Further, knocking out STAT3 in myeloid cells has been shown to cause reduced clinical symptoms in EAE mice [10,18]. Moreover, the inhibition of pathways such as JAK/STAT, which signal through STAT3, has been reported to improve clinical symptoms in different EAE models [40,41,42]. SOCS3 signaling, which inhibits STAT3 activation, has been reported to be decreased in the peripheral blood of MS patients [43]. However, a small-molecule pharmacological inhibition approach to target STAT3 has not yet been explored. STAT3 inhibition may obstruct the downstream signaling of several inflammatory cytokines/mediators which are responsible for the vicious cycle of neuroinflammation. Therefore, in this study, small-molecule inhibitor Stattic was tested to target STAT3 in an RR model of EAE in SJL/J mice.

The Veh + EAE SJL/J group showed a biphasic curve that depicts the relapse and remission of disease symptoms. This is a characteristic feature in SJL/J mice immunized with PLP139-151, which is also observed in human RRMS. This model provides the opportunity to study the effect of an experimental drug on both relapse and remission phases, unlike MOG35-55-induced EAE in C57BL/6 mice, which develops only monophasic disease without relapse [22,44]. We utilized female SJL/J mice, as they develop more severity/incidence than male SJL/L mice. A similar phenomenon is observed in human females, who show a greater prevalence of MS than men [22].

There are different mechanisms that may be responsible for the amelioration of disease symptoms in Stattic-treated mice with EAE. The first mechanism that could be responsible for the amelioration of disease symptoms is impairment in the differentiation of Th0 cells into Th17 cells. Cytokines secreted by antigen-presenting cells can influence the functions of Th0 immune cells and help them differentiate into Th17/Th1/Th2/Treg cells [14,15,45]. It is well known that antigen-presenting cells present autoantigens to Th0 cells in vivo and release polarizing cytokines such as IL-6/IL-21/IL-23, which helps in the differentiation of encephalitogenic Th17 cells [1,6,12,31,32]. These cytokines, i.e., IL-6/IL-21/IL-23, activate STAT3 through cell surface receptors on CD4+ T cells [6,33,46]. Our study showed elevated p-STAT3 levels in CD4+ T cells that were linked with increased IL-17A/IL-23R expression in EAE mice. Previous studies have clearly shown a great contribution of Th17 cells in the development of neuroinflammation in EAE models, as well as MS patients [6,12,16,34]. This investigation, along with previous studies, indicates that STAT3 signaling in lymphoid cells may propel autoimmune inflammation through the modulation of Th17 immune responses in EAE mice. Therefore, in this study, a reduction in the autoinflammation of EAE mice by Stattic could also be partially due to the downregulation of p-STAT3 levels and Th17-related signature markers.

Neutrophils possess complex biological roles, including oxidant generation, cytokine expression, antigen presentation, and neutrophil extracellular traps, which can modulate the immune responses of other immune cells [12,45,47,48]. MPO is one of the most abundant enzymes in phagocytic immune cells, such as neutrophils, and is found in active MS plaques. Individuals with higher MPO expression have increased susceptibility to MS [49]. MPO is mainly secreted from activated neutrophils and has been implicated in immune cell infiltration and BBB impairment during EAE [50,51]. Cytokines, oxidants, and matrix metalloproteinases secreted primarily by neutrophils may damage the BBB and promote leukocyte migration into the brain parenchyma [47,48,51,52,53]. Peripheral myeloid cells and the CNS had elevated p-STAT3 levels in our study that were associated with inflammatory proteins such as iNOS/MPO. MPO and iNOS generate highly reactive molecular entities, such as peroxynitrite, hypochlorous acid, lipid peroxides, and aldehydes, which can amplify neuroinflammatory damage during EAE [42,50,51,54]. Moreover, a recent study has shown that SOCS3 (negative regulator of STAT3) deficiency in myeloid cells is associated with neutrophil infiltration in the CNS and excessive oxidant generation [42]. STAT3 blockade by Stattic caused attenuation in STAT3 activation with a concomitant reduction in oxidative inflammation in peripheral myeloid cells and the CNS. Therefore, attenuation of neutrophilic/CNS oxidative inflammation by STAT3 blockade could also be responsible for the amelioration of disease symptoms in the RR model of EAE.

Inflammatory mediators in the brain were also altered by the inhibition of STAT3 signaling. iNOS, MPO activity, IL-17A, and IL-1 β were elevated in the CNS of EAE mice. These mediators have been previously shown to be elevated in the myeloid/lymphoid cells of the CNS, such as microglial cells, neutrophils, T cells, and macrophages in EAE mice. These inflammatory cytokines/mediators have been shown to be directly or indirectly responsible for neuronal damage and autoinflammation in an RR model of EAE [48,55,56,57]. Stattic administration caused the downregulation of these mediators, which led to an improvement in clinical symptoms in EAE mice. Therefore, it is plausible that the inhibition of STAT3 is responsible for the amelioration of disease symptoms in EAE mice through its action on multiple cells in the CNS.

In summary, our data suggest that STAT3 activation in myeloid and lymphoid immune cells is linked with the expression/release of inflammatory mediators in an RR model of EAE in SJL/J mice. STAT3 inhibition by Stattic caused an amelioration in disease symptoms through a reduction in oxidative and inflammatory markers in myeloid/lymphoid cells and the CNS. Our study demonstrates that a small molecule inhibition approach to target STAT3 could be useful in the alleviation of neuronal/peripheral autoinflammation in RRMS.

## Data Availability

The authors confirm that all data underlying the findings are fully available without restriction. All relevant data are within the paper.

## Supplementary Materials

The following are available online at https://www.mdpi.com/article/10.3390/pharmaceutics13070925/s1, Figure S1: Representative scatter plot of splenic cell suspension and ELISA methodology.
