# Supplementary material for: Pharmacological Inhibition of STAT3 by Stattic Ameliorates Clinical Symptoms and Reduces Autoinflammation in Myeloid, Lymphoid, and Neuronal Tissue Compartments in Relapsing–Remitting Model of Experimental Autoimmune Encephalomyelitis in SJL/J Mice
Source: Pharmaceutics. 2021 Jun 22;13(7):925. doi: 10.3390/pharmaceutics13070925 (PMC8308768; doi:10.3390/pharmaceutics13070925)
Supplement: Supplementary file 1 [file pharmaceutics-13-00925-s001.zip › pharmaceutics-1253160-supplementary.pdf]

pharmaceutics

Supplementary Materials: Pharmacological Inhibition of STAT3 by Stattic Ameliorates Clinical Symptoms and Reduces Autoinflammation in Myeloid, Lymphoid, and Neuronal Tissue Compartments in Relapsing–Remitting Model of Experimental Autoimmune Encephalomyelitis in SJL/J Mice

Khalid Alhazzani, Sheikh F. Ahmad, Naif O. Al-Harbi, Sabry M. Attia, Saleh A. Bakheet, Wedad Sarawi, Saleh A. Alqarni, Mohammad Algahtani and Ahmed Nadeem *

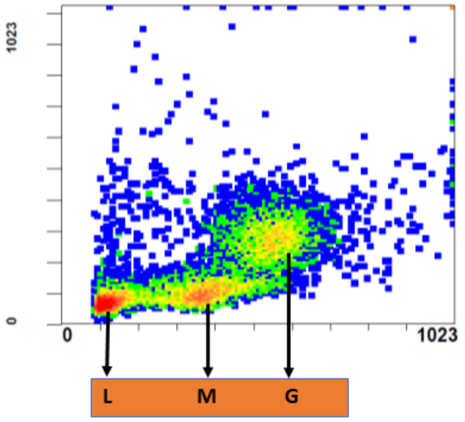

Figure S1. Representative side and forward scatter flow plot of splenic cell suspension of an immunized SJL/J mouse. Splenic cells were immunolabeled and 10,000 events were acquired on a flow cytometer. Based on forward and side scatter, splenic leukocytes were separated into lymphocytes, monocytes, and granulocytes. L, M, and G depict lymphocyte (≈65% of total cells), monocyte (≈20% of total cells) and granulocyte (≈15% of total cells) gates respectively according to conventional gating strategy. % of CD4+ T cells then further identified in Gate L, whereas % of GR-1+ neutrophils were identified in Gate G. Percentage of other intracellular markers (pSTAT3/cytokines/oxidative stress) was then analyzed on CD4+T cells and GR-1+ cells.

Homogenization of brain samples and measurement of cytokines by ELISA

Cerebral cortex was cut out in ice-cold PBS (pH–7.4) with protease inhibitors cocktail followed by homogenization in a Dounce homogenizer. Homogenate was then centrifuged at 12,000 rpm for 20 min in a refrigerated centrifuge. Supernatants were taken out and stored at −80°C before being utilized for ELISA within a month. Protein levels of IL-17A/IL-1β in the brain supernatant samples were measured using a solid-phase sandwich ELISA which is designed to measure the amount of the target bound between a matched antibody pair. IL-17A/IL-1β-specific antibody was pre-coated in the wells of the supplied microplate (R&D Systems, USA; Biolegend, USA). Brain samples, standards, or controls were then added into these wells and allowed to bind to the immobilized (capture) antibody. The sandwich was formed by the addition of the second (detector) antibody followed by addition of a substrate solution that reacts with the enzyme-antibody-target complex to produce measurable signal in the form of colored product. The intensity of colored complex was directly proportional to the concentration of cytokine present in the sample which was determined by a microplate reader (BioTek ELx808, Winooski, VT, USA). Standard curves were generated using the recombinant IL-17A/IL-1β standards.

Protein concentration in the samples was measured using Bradford assay. Cytokines levels were normalized by the protein content of the respective samples. Results are expressed as pg/mg protein.
